# Protective Role of α2HS-Glycoprotein in HBV-Associated Liver Failure

**DOI:** 10.3390/ijms12063846

**Published:** 2011-06-10

**Authors:** Xia-Hong Dai, Pan Zhang, Mei-Fang Xiao, Rong-Rong Zhou, Bao-Xin Zhang, Guan-Sheng Hu, Ze-Bing Huang, Xue-Gong Fan

**Affiliations:** 1 Department of Infectious Diseases, Xiangya Hospital, Central South University, Changsha, Hunan, 410008, China; E-Mails: daixh0630@163.com (X.-H.D.); xmf427@126.com(M.-F.X.); rr_xy@hotmail.com (R.-R.Z.); zhangbx73@sina.com (B.-X.Z.); huguansheng@126.com(G.-S.H.); huangabing0330@yahoo.com.cn (Z.-B.H.); 2 Department of Infectious Diseases, The Third Affiliated Hospital of Xiangya, Central South University, Changsha, Hunan, 410008, China; E-Mail: Zhanghy@126.com

**Keywords:** human fetuin-a, α2-Heremans-Schmid glycoprotein, spermine, acute-on-chronic liver failure

## Abstract

In this study, levels of plasma α2-Heremans-Schmid glycoprotein, serum tumor necrosis factor-α, serum liver function parameters and short-term mortality were measured in 100 hepatitis B patients. Release of interleukin-6 and tumor necrosis factor-α from the lipopolysaccharide-stimulated peripheral blood mononuclear cells in the presence/absence of spermine and α2-Heremans-Schmid glycoprotein were analyzed by enzyme-linked immunosorbent assay to determine the significance and potential mechanism of α2-Heremans-Schmid glycoprotein in hepatitis B virus-associated liver damage. Results showed that serum α2-Heremans-Schmid glycoprotein levels in acute-on-chronic liver failure patients were significantly lower than that in chronic hepatitis B patients or healthy controls (p < 0.05). A negative dependence between serum human α2-Heremans-Schmid glycoprotein and tumor necrosis factor-α levels was observed. Interleukin-6 and tumor necrosis factor-α levels in the lipopolysaccharide-induced peripheral blood mononuclear cell supernates were significantly reduced by spermine and/or α2-Heremans-Schmid glycoprotein. The latter two proteins jointly inhibited cytokine release. These observations suggest that plasma α2-Heremans-Schmid glycoprotein is an independent marker of liver damage and a prognostic indicator of hepatitis B virus chronicity. It may reduce liver inflammation by partially inhibiting release of inflammatory factors from activated peripheral blood mononuclear cells.

## 1. Introduction

Fetuin-a is a 60 kDa glycoprotein first identified by Pederson *et al.* in 1944. Human fetuin-a, now known as α2-Heremans-Schmid glycoprotein (AHSG), is expressed in several tissues of the embryo [1]. Although the principal source of this protein in adults are the hepatocytes [2], it is also expressed by monocytes or macrophages. AHSG is a natural antagonist of several growth factors, such as insulin, transforming growth factor-â, epidermal growth factor, lymphocyte mitogenic protein, and hepatocyte growth factor/scatter factor [3,4]. The main physiological functions of AHSG have been widely studied and demonstrated [5–7]. Recently, interest has been focused on the anti-inflammatory effect of this protein. As a negative acute-phase protein, its normal circulating level in an adult is significantly reduced during injury and infection. Wang *et al.* observed that spermine, a ubiquitous biogenic polyamine that accumulates at sites of injury or inflammation, could inhibit macrophage cytokine synthesis in the presence of AHSG [8]. In addition, in vitro and in vivo data have indicated that AHSG suppresses the release of tumor necrosis factor (TNF) from lipopolysaccharide (LPS)-stimulated macrophages [7]. AHSG plays a protective role in experimental sepsis by attenuating late mediators of lethal systemic inflammation, and protects the fetus from TNF [9,10].

Hepatitis B is still a major health problem. Globally, there are around 350 million people infected with chronic hepatitis B virus (HBV), 75% of which are in the Asia-Pacific region. Immune dysfunction is the main pathogenic mechanism in the chronicity of this infection and HBV-related liver failure [11]. Many lines of evidence have suggested that cellular and humoral immune responses are required for viral clearance, and that non-HBV-specific immune response is responsible for the liver damage [12–14]. Recent data from animal models and humans indicate that excessive stimulation of the endotoxin, which is usually produced by intestinal tract bacteria in patients with hepatitis B, results in secondary live injury and aggravates hepatitis.

Considering the anti-inflammatory role of AHSG and the excessive immune responses in the pathology of hepatitis B, we speculated that AHSG may be involved in reducing secondary hepatic injury by inhibiting excessive inflammation in patients with viral hepatitis and/or liver failure. Thus, serum indicators of liver function, such as plasma AHSG and TNF-α level in subjects with chronic hepatitis B and acute-on-chronic liver failure (ACLF) were examined to reveal the clinical significance of AHSG. To investigate the potential mechanism of this protein, we hypothesized that in the intestinal endotoxemia-correlated progression of ACLF, AHSG may partially regulate the release of inflammatory mediators such as TNF-α and interleukin 6 (IL-6) by inhibiting the activation of primary human peripheral blood mononuclear cells (PBMCs). Thus, we cultured human PBMCs and measured the TNF-α and IL-6 levels *in vitro* to investigate the potential mechanism of AHSG in inhibiting increased LPS-induced inflammation.

## 2. Results

### 2.1. Subjects

One hundred hospitalized patients with chronic hepatitis B (CHB) and HBV-related ACLF were included in the present study (Table 1). According to the recommendations of the Asian Pacific Association for the Study of the Liver [15], diagnosis of CHB and ACLF was based on clinical manifestations and elevated values of serum alanine aminotransferase (ALT), total serum bilirubin (TBIL), and prothrombin time activity (PTA) percentage for at least one year after the initial determination associated with positive serum HBV markers [16]. There was no evidence of co-infection with other hepatotropic viruses. Other possible causes of liver damage, such as alcohol, drugs or autoimmune diseases, and genetic liver diseases were excluded. Patients who had received immunomodulator therapy within three months were also excluded. As controls, 12 healthy individuals were recruited. The study was approved by the hospital ethics committee, and all patients and controls gave their consent before blood donation.

### 2.2. Serum AHSG and TNF-α in Subject with CHB and ACLF

**Figure 1:**
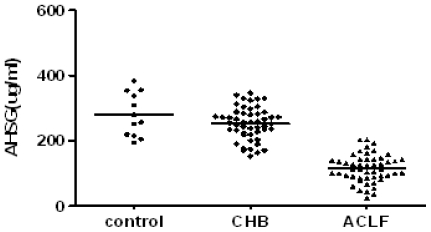
Serum AHSG level in patients and controls. *P* < 0.01, serum AHSG level was significantly lower in ACLF patients than that in CHB patients and healthy controls.

**Figure 2:**
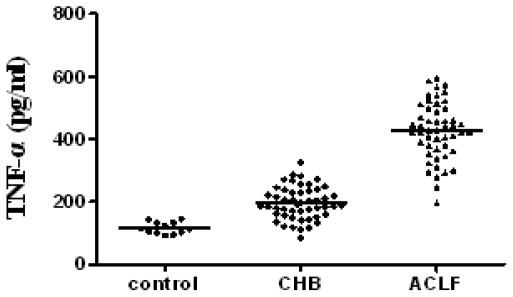
Serum TNF-α level in patients and controls. *P* < 0.01 Serum TNF-α level was significantly higher in ACLF patients than in CHB patients and healthy controls.

The mean concentrations of AHSG and TNF-α in the sera of subjects with CHB and HBV-related ACLF are presented in Figures 1 and 2.

The serum AHSG level was significantly lower in ACLF patients (115.21 ± 42.28 μg/mL) than that in CHB patients (253.02 ± 49.56 μg/mL, *P* < 0.01) and healthy controls (280.33 ± 66.13 μg/mL, *P* < 0.01), respectively. However, there were no significant differences between CHB patients and the healthy controls (*P* > 0.05). The plasma TNF-α level in ACLF patients (426.71 ± 91.37 pg/mL) was significantly higher than in healthy controls (116.50 ± 18.76 pg/mL) and CHB patients (196.22 ± 52.16 pg/mL) (*P* < 0.01).

### 2.3. Serum AHSG Levels and Short-Term Mortality

The fifty ACLF patients were followed up for one month. Fifteen of them died due to liver failure (group A); whereas the rest showed improvement in clinical and laboratory tests (group B). The serum AHSG levels (*P* < 0.05) and PTA (*P* < 0.01) in group B were significantly higher than those in group A (Table 2). However, there was no significant difference in the TBIL, serum albumin, and TNF-α levels (*P* > 0.05) between the two groups.

### 2.4. Correlation Analysis

**Figure 3:**
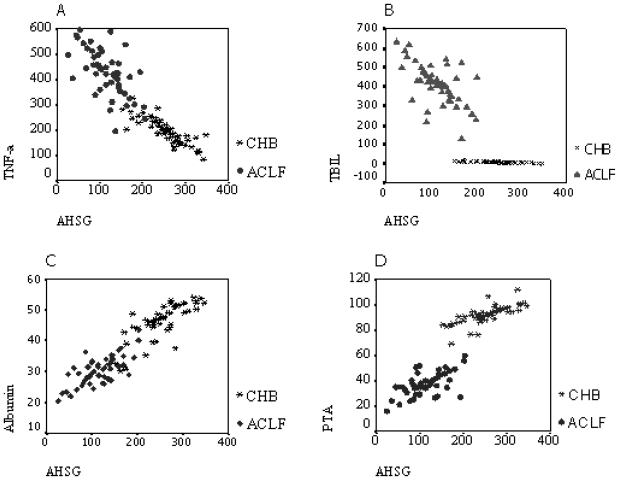
Correlations between serum AHSG levels and laboratory parameters in CHB and ACLF patients. **(A)** TNF-α; **(B)** TB1IL; **(C)** Albumin; **(D)** PTA. *P* < 0.01 serum ANSG level was positively correlated with PTA or serum albumin level, while negatively correlated with serum TNF-α level or TBIL concentration in both CHB and ACLF patients.

To investigate whether plasma AHSG was associated with the circulating markers of inflammation and liver function *in vivo*, we analyzed cross-sectional data from 100 subjects, which consisted of 50 CHB patients and 50 ACLF patients. As shown in Figure 3, a positive correlation was observed between the serum AHSG level and PTA or serum albumin. A negative correlation was found between serum AHSG and TNF-α levels or serum TBIL concentration.

### 2.5. Effects of AHSG on Cytokine Production in LPS-Stimulated PBMCs

Cells were cultured in seven groups. Culture media were collected after 24 h and assayed for TNF-α and IL-6. The results are illustrated in Table 3. Cultures exposed to LPS alone resulted in a significant increase in the TNF-α level from 6.44 ± 3.88 (group 1) to 423.73 ± 97.66 pg/mL (group 2) (*P* < 0.01), and in the IL-6 level from 19.21 ± 5.59 (group 1) to 584.73 ± 82.36 pg/mL (group 2) (*P* < 0.01). However, addition of spermine (group 3) significantly reduced the TNF-α and IL-6 levels to 72.97 ± 18.32 and 239.96 ± 48.26 pg/mL, respectively (*P* < 0.01). Substantial decreases in the TNF-α and IL-6 levels were observed when AHSG and spermine were added simultaneously (group 4) (*P* < 0.05).

To antagonize the effect of endogenous AHSG on the PBMCs, an AHSG-specific antibody was administered to the LPS-stimulated PBMCs in the presence of spermine (group 5). The TNF-α and IL-6 levels in group 5 increased to 261.43 ± 54.07 and 555.42 ± 69.78 pg/mL, respectively, which was significantly higher than those in groups 3 or 4 (*P* < 0.01). However, the level of TNF-α in group 5 was still significantly lower than that in the LPS-stimulated group (group 2, *P* < 0.01), while no significant difference of IL-6 level was observed between group 5 and group 2 (P > 0.05).

Serum albumin was chosen as a protein control to investigate the specificity of the anti-inflammatory effect of AHSG. The addition of albumin (group 6) significantly decreased the TNF-α or IL-6 levels. On the other hand, exposure to AHSG alone produced approximately 258.55 ± 54.87 pg/mL of TNF-α and 503.78 ± 90.87 pg/mL of IL-6 after 24 h, which were significantly lower than those in group 2 (*P* < 0.05). However, no significant difference was observed in TNF-α or IL-6 levels in groups 5, 6, and 7 (*P* > 0.05). Thus, the production of TNF-α and IL-6 byLPS-stimulated human PBMC could be reduced by a given dose of AHSG and spermine.

## 3. Discussion

AHSG, a member of the cystatin super family [1], is involved in various conditions such as metabolic disease, tumor, and sepsis [17,18]. Variations of this protein have been confirmed in patients with liver diseases [19–21]. Low serum AHSG levels are observed in patients with alcoholic hepatitis, acute drug-induced hepatitis, and chronic autoimmune hepatitis, as well as in fatty liver, primary biliary cirrhosis, and hepatocellular cancer patients. After long-term (up to 1 year) follow up, Kalabay *et al.* concluded that the sensitivity, specificity, and predictive values of serum AHSG concentration exceeded the scores from the model for end-stage liver disease. However, the potential role of AHSG in patients with viral hepatitis remains unknown. Our present study demonstrated a significant decline in the serum AHSG levels in ACLF patients compared with those in CHB patients or healthy controls. Further analysis showed a negative correlation between the AHSG and serum TNF-α level. Analysis of the relationship between the serum AHSG levels and short-term mortality showed that serum AHSG level was significantly higher in the surviving patients compared with those who succumbed to their illness; this reveals that low serum AHSG may be responsible for poor prognosis. A positive correlation of the serum AHSG level with conventional liver function indicators (PTA and serum albumin levels) and a negative correlation with TBIL concentration were observed. These findings indicate that the serum AHSG level was directly linked to the severity of liver damage. Hence, serum AHSG level is a new independent marker of liver damage in chronic HBV-infected patients. AHSG may reduce excessive liver inflammation and cell necrosis by inhibiting the TNF-α expression. The reduction of serum AHSG level in viral hepatitis may be explained by the following: First, the synthesis of AHSG is reduced significantly due to the large number of liver cells undergoing apoptosis or necrosis, and concomitant loss of function of residual liver cells. Second, substantial amounts of AHSG may be consumed in the inhibition of increased expression of cytokines (such as TNF-α) during liver failure. These hypotheses need to be proved by further studies.

Besides, significantly lower serum AHSG level in deceased ACLF patients was observed than in survived patients, while no significant differences in serum TNF-α, TBIL and albumin levels were observed between the two groups. However, negative correlations between serum AHSG level and TNF-α/TBIL/albumin level in ACLF patients were observed during further analysis. The possible reasons for the discrepancy were as follows: (i) the correlation coefficients were not big enough. Correlation coefficients between serum AHSG and TNF-α/TBIL/albumin levels were (−0.622, *P* < 0.05), (−0.560, *P* < 0.05) and (0.572, *P* < 0.05), respectively. Therefore, apparent statistical differences haven’t been observed; (ii) the comparatively small sample size (subjects of ACLF = 50, survived patients = 35, deceased ACLF patients = 15) might be another reason.

Hepatic and circulating inflammatory cytokines play a critical role in the pathophysiology of ACLF [22]; the importance of intestinal endotoxin in this process has drawn more attention [23]. The present study suggests that the LPS-macrophage activation-TNF-α-axis [24] is the core of secondary liver injury leading to liver failure. TNF-α and IL-6 are considered critical inflammatory cytokines in immunopathogenesis. Although various agents can cause hepatocyte injury and liver failure, many studies in patients and animal models have strongly suggested that TNF-α and IL-6, the major mediators of the acute-phase response, are involved in the induction of apoptosis and in triggering liver damage, which ultimately lead to hepatic failure [25]. Therefore, the removal of proinflammatory cytokines from the plasma might be help to alleviate this condition [26]. Thus, we targeted TNF-α and IL-6 to reveal the potential mechanism by which AHSG may be involved in the pathophysiology of HBV-related ACLF. Our *in vitro* investigation showed that the levels of these cytokines increased significantly in the LPS-stimulated PBMCs, which was consistent with previous studies [27,28]. In contrast, the IL-6 and TNF-α levels were significantly reduced when spermine was added; this indicates that spermine inhibited the release of these inflammatory mediators in the presence of endogenous AHSG. We also found that AHSG had a synergistic effect on inhibiting the release of TNF-α and IL-6 when added with spermine. A combination of exogenous spermine and AHSG maximally reduced the release of the inflammatory mediators.

When an AHSG antibody was added to antagonize the effect of endogenous AHSG, significant increases in the TNF-α and IL-6 levels were observed, revealing that the AHSG antibody antagonized not only the contribution of endogenous AHSG on the surface of the PBMCs, but also the anti-inflammatory effect of spermine. This behavior confirmed that AHSG was essential for spermine. The same result has been obtained in previous studies wherein AHSG alone administered at low concentrations did not suppress the release of inflammatory mediators. However, AHSG itself could also suppress the release of many inflammatory mediators such as nitric oxide and HMGB1 [29] when administered at high concentrations.

Thus, we consider that in the process leading to its anti-inflammatory effect, AHSG plays a protective role by attenuating the release of pro-inflammatory mediators. In addition, serum albumin, a negative acute-phase protein and an inflammatory marker, which has the similar molecular weight and is also synthesized in the liver, was chosen as the control. Albumin suppressed the inflammatory response to some extent. Although the mechanism for this process remains ambiguous, it may be related to: (1) the change in the protein proportion in the media which favors cell proliferation in the presence of albumin; (2) a potential anti-inflammatory role of albumin, which needs to be proved; and (3) in the presence of albumin the concentration of free LPS is reduced due to its binding to albumin. This would directly result in the decreased stimulating ability of LPS.

In summary, low serum AHSG level and intestinal endotoxemia are critical pathological states in subjects of liver failure, while the peripheral blood mononuclear cells and inflammatory factors such as TNF-α and IL-6 are important immune targets in the pathological process of liver failure. Focusing on the *in vivo* situations above, we studied the effect of administrated AHSG on the LPS stimulated release of inflammatory factors from PBMCs *in vitro*. The results above indicated that certain dose of AHSG attenuated the release of TNF-α and IL-6 from LPS stimulated PBMCs. Hence, we consider that there may be a vicious cycle in the pathologic progression of chronic severe hepatitis, especially ACLF. The synthesis of AHSG is reduced significantly because of liver damage and cell necrosis, which results in a reduction of the anti-inflammatory effect of circulating AHSG. Thus, the hepatic and circulating inflammatory cytokines are produced in large amounts and mediate further damage to the liver. Therefore, administration of exogenous AHSG may be beneficial to reconstitute liver function. The present study revealed the potential role and preliminary data on the mechanism of AHSG in HBV-associated liver failure. The study had limitations in the sample size; therefore, further studies should be designed to clarify the specific mechanisms.

## 4. Materials and Methods

### 4.1. Determination of Serum AHSG and TNF-α Concentration

Blood samples were collected from patients with empty stomachs in luce prima. Enzyme-linked immunosorbent assay (ELISA) for serum levels of AHSG (Bio Vendor RD191037100, Brno, Czech Republic) and TNF-α (Abazyme EL10019, Shanghai, China) were performed according to manufacturer instructions.

### 4.2. Preparation and Culture of PBMCs

PBMCs (10 mL, ~1 × 10^6^ cell/mL; 3 replicates) were isolated from the peripheral blood of an adult male healthy donor by Ficoll-Hypaque density gradient centrifugation (once a week for three weeks, three replicates for every time). Around 1 × 10^6^ cells/mL were suspended in RPMI 1640 (Gibco, Grand Island, NY, USA) culture medium supplemented with 10% heat-inactivated fetal calf serum (Gibco, Bethesda, MD, USA), 100 U/mL penicillin, and 100 mg/mL streptomycin (Huamei Co., Shanghai, China). Cell viability was determined by trypan blue exclusion (>95% viable).

### 4.3. Cell Culture and Cytokine ELISA

PBMCs (1 × 10^6^ cell/mL) were cultured in 24-well polystyrene plates (Costar, Newark, NJ, USA) at 37 °C in the presence of 5% CO^2^ for 4 h. Each well was additionally supplemented with 1.0 mL of the complete medium containing one or a combination of the following: 50 μg/mL fetuin-a (Sigma-Aldrich Co., St. Louis, MO, USA), 50 μmol/L spermine (Sigma-Aldrich Co., St. Louis, MO, USA), and 50 μg/mL AHSG antibody (Abcam, San Francisco, CA, USA) (Table 4). Some wells were instead supplemented with sterile bovine serum albumin (Gibco, Grand Island, NY, USA). A 100 ng/mL standard dose of LPS (Sigma-Aldrich Co., St. Louis, MO, USA) was added to the wells, which were used for experiments on LPS stimulation. After 24 h incubation under 5% CO^2^ at 37 °C, the culture media were collected, and the TNF-α and IL-6 levels were measured by ELISA (SUNBIO HE001, SUNBIO HE003, Beijing, China) according to the instructions of the manufacturer.

### 4.4. Statistical Analysis

All data were analyzed using SPSS 11.5 software and were summarized as means and standard deviations. The hypothesis was tested using least-significant-difference *t*-test, and linear correlation analysis was used between two sets of data. ANOVA analysis was used for the comparison of different groups. For all tests, a two-sided probability (P < 0.05) was considered statistically significant.

## 5. Conclusions

Serum AHSG could be considered as a new independent marker of liver damage in chronic viral hepatitis, especially ACLF. Increased administration of AHSG may be beneficial in reducing liver damage, an effect which is worthy of further study.

## Figures and Tables

**Table 1:** Clinical characteristic of the studied subjects ( *X̄* ± SD).

	Controls	CHB	ACLF
**Sex (M/F)**	12 (8/4)	50 (37/13)	50 (38/12)
**Average Age (yr)**	38.3 ± 9.5	40.3 ± 5.8	39.0 ± 6.0
**Average ALT (U/L)**	21.96 ± 4.72	55.48 ± 10.60	449.21 ± 57.40
**Average TBIL (μmol/L)**	8.42 ± 1.40	9.81 ± 3.64	414.08 ± 102.63
**Average A (g/L)**	44.65 ± 5.39	46.03 ± 6.18	30.28 ± 4.65
**PTA (%)**	93.34 ± 2.13	92.00 ± 7.39	37.60 ± 9.13
**Serum HBV-DNA (+)**	0	30	50

CHB, chronic hepatitis B; ACLF, HBV-related acute-on-chronic liver failure; ALT, alanine aminotransferase; TBIL, total serum bilirubin; A, serum albumin; PTA, prothrombin activity.

**Table 2:** Correlation between serum AHSG levels and short-term mortality.

Groups	n	AHSG (μg/mL)	PTA (%)	TBIL (μmol/L)	A (g/L)	TNF-α (pg/mL)
A	15	85.26 ± 20.54#	29.9 ± 8.22*	453.09 ± 117.98	28.53 ± 4.85	448.27 ± 100.58
B	35	128.05 ± 36.52	40.90 ± 7.26	397.36 ± 92.13	31.03 ± 4.43	417.47 ± 87.03

Compared with group B,

* *P* < 0.01;

# *P* < 0.05.

**Table 3:** Effects of AHSG on PBMC TNF-α and IL-6 levels.

Group		Supernatant TNF-α levels (pg/mL)	Supernatant IL-6 levels (pg/mL)
1	PBMC	6.44 ± 3.88	19.21 ± 5.59
2	PBMC + LPS	423.73 ± 97.66*	584.73 ± 82.36&
3	PBMC + LPS + spermine	72.97 ± 18.32**	239.96 ± 48.26**
4	PBMC + LPS + spermine + AHSG	25.11 ± 5.61***	114.90 ± 30.83***
5	PBMC + LPS + spermine + AHSG antibody	261.43 ± 54.07#	555.42 ± 69.78@
6	PBMC + LPS + albumin	223.92 ± 46.65##	403.46 ± 78.80##
7	PBMC + LPS + AHSG	258.55 ± 54.87###	503.78 ± 90.87###

* *P* < 0.01, *versus* groups 1, 3, 4, 5, 6 and 7;

** *P* < 0.05, *versus* groups 1, 2, 4, 5 and 6;

*** *P* < 0.01, *versus* groups 2, 3, 5 and 6;

# *P* < 0.01, *versus* groups 1, 2, 3 and 4;

## *P* < 0.05, *versus* groups 1, 2, 3 and 4;

### *P* < 0.05, *versus* groups 1, 2, 3, and 4;

& *P* < 0.01, *versus* groups 1, 3, 4, 6;

@ *P* < 0.05, *versus* groups 1, 3 and 4.
